# Air Pollution and Health: Bridging the Gap from Sources to Health Outcomes

**DOI:** 10.1289/ehp.1103660

**Published:** 2011-04

**Authors:** Paul A. Solomon

**Affiliations:** U.S. Environmental Protection Agency, Office of Research and Development, Las Vegas, Nevada, E-mail: solomon.paul@epamail.epa.gov

The U.S. Environmental Protection Agency (EPA) has established National Ambient Air Quality Standards (NAAQS) for six principal air pollutants (“criteria” pollutants): carbon monoxide (CO), lead (Pb), nitrogen dioxide (NO_2_), particulate matter (PM) in two size ranges [< 2.5 μm (PM_2.5_) and < 10 μm (PM_10_)], ozone (O_3_), and sulfur dioxide (SO_2_) (U.S. EPA 2010b). Although associations have been identified between these pollutants and adverse health effects, considerable uncertainty remains regarding *a*) methods and approaches to understanding relationships between air pollution and health effects; *b*) which components (gas and/or aerosol) and sources are most toxic; *c*) the mechanisms of actions of the pollutants and causal relationships; *d*) effect of confounding factors, and *e*) which populations are susceptible {U.S. EPA 2006a (Pb), 2006b (O_3_), 2008a [NO_x_ Integrated Science Assessment (ISA)], 2008b (SO_x_ ISA), 2009 (PM), 2010 (CO)}. This holds true especially for PM, because it is composed of many components with significant spatial and temporal variation (U.S. EPA 2009). Air pollution and health research continues to reduce these uncertainties across the source-to-health effects paradigm as described by the National Research Council (NRC) *Research Priorities for Airborne Particulate Matter,* volumes I–IV, (NRC 1998, 1999, 2001, 2004) and the U.S. EPA (2006a, 2006b, 2008a, 2008b, 2009b, 2010a).

Linking air pollution and adverse health effects is complicated and requires expertise across a range of scientific disciplines—from atmospheric to exposure to health sciences, as well as inclusion of air quality managers and policy makers who implement and develop policy to reduce risk from air pollution. Interaction among these groups at different points in time helps to identify gaps in knowledge and suggest future research directions. One such opportunity was the international specialty conference “Air Pollution and Health: Bridging the Gap from Sources to Health Outcomes,” sponsored by the American Association for Aerosol Research (AAAR 2010). The conference, chaired by myself and Maria Costantini (Health Effects Institute), was designed to help disseminate and integrate results from scientific studies that cut across the range of air pollution– and health-related disciplines of the source-to-health effects continuum. The conference addressed the science of air pollution and health within a multipollutant framework, focusing on five key science areas—sources, atmospheric sciences, exposure, dose, and health effects—as identified by the NRC (1998). Eight key policy-relevant science questions that integrated across various parts of these science areas formed the basis of the meeting, and a ninth question addressed the policy implications of the findings (see Appendix).

This was the AAAR’s third international specialty conference and extended the findings presented at the AAAR’s first specialty conference “Particulate Matter: Atmospheric Sciences, Exposure, and the Fourth Colloquium on PM and Human Health,” held in Pittsburgh, Pennsylvania, in 2003 (Davidson et al. 2005).

Results from the 2010 AAAR Air Pollution and Health conference are being published in *Environmental Health Perspectives* (*EHP*); *Air Quality, Atmosphere and Health; Aerosol Science and Technology; Atmospheric Environment*; and *Inhalation Toxicology* (Solomon 2010).

This issue of *EHP* includes conference papers on the importance of a multipollutant approach and of individual components of particulate matter to understanding linkages between sources and adverse health outcomes, including respiratory and/or cardiovascular diseases (Ito et al. 2011; Lall et al. 2011; Rohr et al. 2011; Spira-Cohen et al. 2011; Zhou et al. 2011), associated effects, such as inflammation (Alexeeff et al. 2011), and birth outcomes associated with exposures to traffic-related pollution during gestation (Malmqvist et al. 2011). Several air pollution components and sources were evaluated, including elemental carbon and secondary organic aerosol, traffic, local industrial sources, and residential oil and wood burning. Where studied, some effects varied by season and location over sufficient time (specifically, Detroit, MI; Seattle, WA; New York, NY), likely due to the influence of different source impacts. In addition, this issue includes a review of population characteristics related to susceptibility (Sacks et al. 2011), and an accountability study of the feasibility of hybrid regional–local modeling to assess health improvements in small communities (Lobdell et al. 2011).

## Figures and Tables

**Figure:**
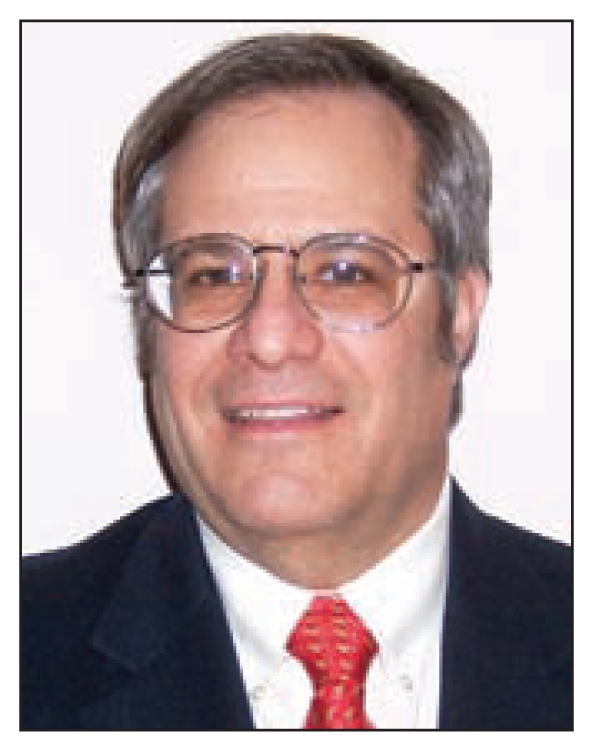
Paul A. Solomon

## Appendix

- How does our understanding of the health effects of air pollutants (singly or in mixtures) help identify pollutants that can be linked to sources the control of which would provide maximal health benefits? (overarching theme)
- How reliable are methods (measurements and models) and approaches (epidemiological and toxicological) for studying and quantifying the links between air pollutants (species and or sources) and adverse health effects?
- How do relevant pollutant properties vary in space and time from sources and in ambient air? What are the implications of these variations for population exposure?
- What advances have been made in understanding the relationships between exposure, both spatially and temporally, and estimates of dose that tie to health outcomes?
- Are patterns emerging that relate component(s) of air pollution and/or source types to mechanisms? What is the status of identifying and measuring biomarkers of exposure and/or adverse health effects from air pollution?
- Who are the susceptible populations? What drives different susceptibilities to the same or different air pollutants? Are there susceptibility traits associated with specific health outcomes that are common among the subpopulations?
- What roles do confounding or other factors have in increasing, decreasing, or obscuring attribution of the true health effects from ambient air pollutants?
- Do actions taken to improve air quality result in reduced ambient concentrations of relevant pollutants, exposure, and health effects? Have we encountered unintended consequences?
- What are the policy implications of our improved understanding of the source to health effect paradigm?
